# Ethyl 5-methyl-4-oxo-3-phenyl-2-propyl­amino-3,4-dihydro­thieno[2,3-*d*]pyrimidine-6-carboxyl­ate

**DOI:** 10.1107/S1600536809033595

**Published:** 2009-08-29

**Authors:** Ai-Hua Zheng, Yan-Mei Ren, Jing Xu

**Affiliations:** a Institute of Medicinal Chemistry, Yunyang Medical College, Shiyan 442000, People’s Republic of China; b Clinical Laboratory, Zhushan Center for Disease Control and Prevention, Shiyan 442000, People’s Republic of China

## Abstract

The title compound, C_19_H_21_N_3_O_3_S, was synthesized *via* the aza-Wittig reaction of functionalized imino­phospho­rane with phenyl isocyanate under mild conditions. In the mol­ecule, the fused thienopyrimidine ring system is essentially planar, with a maximum deviation of 0.072 (2) Å, and makes a dihedral angle of 60.11 (9)° with the phenyl ring. An intra­molecular C—H⋯O hydrogen bond is present. The crystal packing is stabilized by inter­molecular N—H⋯O and C—H⋯O hydrogen bonds.

## Related literature

For the preparation and biological and pharmaceutical activities of pyrimidinone derivatives, see: Modica *et al.* (2004 ▶); Panico *et al.* (2001 ▶). For the biological activity of thienopyrimidine derivatives, see: Ding *et al.* (2004 ▶).
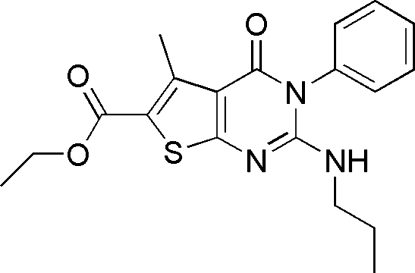


## Experimental

### 

#### Crystal data

C_19_H_21_N_3_O_3_S
*M* *_r_* = 371.45
Orthorhombic, 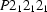

*a* = 8.1682 (2) Å
*b* = 14.1247 (3) Å
*c* = 16.0672 (5) Å
*V* = 1853.73 (8) Å^3^
*Z* = 4
Mo *K*α radiation
μ = 0.20 mm^−1^
*T* = 298 K
0.16 × 0.12 × 0.10 mm

#### Data collection

Bruker SMART 4K CCD area-detector diffractometer
Absorption correction: multi-scan (*SADABS*; Sheldrick, 1996 ▶) *T*_min_ = 0.969, *T*_max_ = 0.980
10064 measured reflections
4472 independent reflections
4226 reflections with *I* > 2σ(*I*)
*R*_int_ = 0.031

#### Refinement

*R*[*F* ^2^ > 2σ(*F* ^2^)] = 0.053
*wR*(*F* ^2^) = 0.136
*S* = 1.13
4472 reflections
241 parameters
H atoms treated by a mixture of independent and constrained refinement
Δρ_max_ = 0.39 e Å^−3^
Δρ_min_ = −0.37 e Å^−3^
Absolute structure: Flack (1983 ▶), 1861 Freidel pairs
Flack parameter: 0.08 (10)

### 

Data collection: *SMART* (Bruker, 2001 ▶); cell refinement: *SAINT-Plus* (Bruker, 2001 ▶); data reduction: *SAINT-Plus*; program(s) used to solve structure: *SHELXS97* (Sheldrick, 2008 ▶); program(s) used to refine structure: *SHELXL97* (Sheldrick, 2008 ▶); molecular graphics: *PLATON* (Spek, 2009 ▶); software used to prepare material for publication: *SHELXTL* (Sheldrick, 2008 ▶).

## Supplementary Material

Crystal structure: contains datablocks I, global. DOI: 10.1107/S1600536809033595/at2864sup1.cif

Structure factors: contains datablocks I. DOI: 10.1107/S1600536809033595/at2864Isup2.hkl

Additional supplementary materials:  crystallographic information; 3D view; checkCIF report

## Figures and Tables

**Table 1:** Hydrogen-bond geometry (Å, °)

*D*—H⋯*A*	*D*—H	H⋯*A*	*D*⋯*A*	*D*—H⋯*A*
C6—H6⋯O2^i^	0.93	2.58	3.359 (3)	142
C2—H2⋯O2^ii^	0.93	2.50	3.432 (3)	177
N3—H3*A*⋯O1^iii^	0.88 (3)	2.08 (3)	2.863 (3)	147 (3)
C16—H16*C*⋯O2	0.96	2.31	3.000 (3)	128

Symmetry codes: (i) 
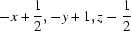
; (ii) 
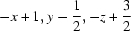
; (iii) 
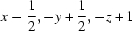
.

**Fig. 1.**
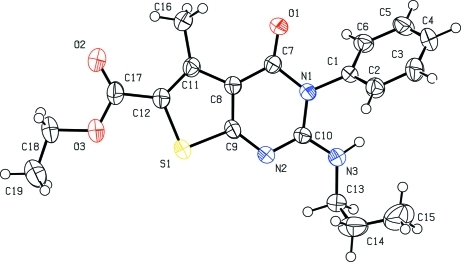
ORTEP drawing and atom labelling scheme of the title compound with thermal ellipsoids drawn at the 50% probability level.

## supplementary crystallographic information

### Comment

The derivatives of thienopyrimidine are of great importance because of their remarked biological properties (Ding *et al.*, 2004). We have recently focused on the synthesis of fused heterocyclic systems containing a fused pyrimidinone ring moiety using aza-Wittig reaction. The title compound, may be used as a new precursor for obtaining bioactive molecules and its structure is reported here, Fig.1. The bond lengths and angles are unexceptional. The thienopyrimidinone rings are closer to coplanarity with maximum deviations 0.072 (2)Å and -0.058 (2)Å for C10 and N1, respectively. The phenyl ring is twisted with respect to the pyrimidinone ring, with a dihedral angle of 60.11 (9)°. Intramolecular C—H···O and intermolecular C—H···O, N—H···O hydrogen bonds interactions are present, which stabilize the conformation of the molecule and the crystal structure (Table 1).

### Experimental

To a solution of diethyl 5-((phenylimino)methyleneamino)- 3-methylthiophene-2,4-dicarboxylate(3 mmol) in anhydrous dichloromethane (15 ml) was added propan-1-amine (3 mmol). After stirring the reaction mixture for 1 h, the solvent was removed and anhydrous ethanol (10 ml) with several drops of EtONa in EtOH was added. The mixture was stirred for 5 h at room temperature. The solution was concentrated under reduced pressure and the residue was recrystallized from ethanol to give the title compound in a yield of 78%. Crystals suitable for single-crystal X-ray diffraction were obtained by recrystallization from a mixed solvent of ethanol and dichloromethane (1:1 *v*/*v*) at room temperature.

### Refinement

All H-atoms were positioned geometrically and refined using a riding model with C—H = 0.93 Å, *U*_iso_=1.2*U*_eq_ (C) for C*sp*^2^, N—H = 0.88 Å, *U*_iso_=1.2*U*_eq_ (N) for NH, C—H = 0.97 Å, *U*_iso_ = 1.2*U*_eq_ (C) for CH_2_, C—H = 0.96 Å, *U*_iso_ = 1.5*U*_eq_ (C) for CH_3_.

### Crystal data

**Table:**

C_19_H_21_N_3_O_3_S	*F*(000) = 784
*M**_r_* = 371.45	*D*_x_ = 1.331 Mg m^−^^3^
Orthorhombic, *P*2_1_2_1_2_1_	Mo *K*α radiation, λ = 0.71073 Å
Hall symbol: P 2ac 2ab	Cell parameters from 4659 reflections
*a* = 8.1682 (2) Å	θ = 2.5–28.0°
*b* = 14.1247 (3) Å	µ = 0.20 mm^−^^1^
*c* = 16.0672 (5) Å	*T* = 298 K
*V* = 1853.73 (8) Å^3^	Block, colourless
*Z* = 4	0.16 × 0.12 × 0.10 mm

### Data collection

**Table:**

Bruker SMART 4K CCD area-detector diffractometer	4472 independent reflections
Radiation source: fine-focus sealed tube	4226 reflections with *I* > 2σ(*I*)
graphite	*R*_int_ = 0.031
φ and ω scans	θ_max_ = 28.3°, θ_min_ = 1.9°
Absorption correction: multi-scan (*SADABS*; Sheldrick, 1996)	*h* = −10→5
*T*_min_ = 0.969, *T*_max_ = 0.980	*k* = −18→18
10064 measured reflections	*l* = −21→21

### Refinement

**Table:**

Refinement on *F*^2^	Secondary atom site location: difference Fourier map
Least-squares matrix: full	Hydrogen site location: inferred from neighbouring sites
*R*[*F*^2^ > 2σ(*F*^2^)] = 0.053	H atoms treated by a mixture of independent and constrained refinement
*wR*(*F*^2^) = 0.136	*w* = 1/[σ^2^(*F*_o_^2^) + (0.0772*P*)^2^ + 0.1133*P*] where *P* = (*F*_o_^2^ + 2*F*_c_^2^)/3
*S* = 1.13	(Δ/σ)_max_ < 0.001
4472 reflections	Δρ_max_ = 0.39 e Å^−^^3^
241 parameters	Δρ_min_ = −0.37 e Å^−^^3^
0 restraints	Absolute structure: Flack (1983), 1861 Freidel pairs
Primary atom site location: structure-invariant direct methods	Flack parameter: 0.08 (10)

### Special details

**Table:**

Geometry. All e.s.d.'s (except the e.s.d. in the dihedral angle between two l.s. planes) are estimated using the full covariance matrix. The cell e.s.d.'s are taken into account individually in the estimation of e.s.d.'s in distances, angles and torsion angles; correlations between e.s.d.'s in cell parameters are only used when they are defined by crystal symmetry. An approximate (isotropic) treatment of cell e.s.d.'s is used for estimating e.s.d.'s involving l.s. planes.
Refinement. Refinement of *F*^2^ against ALL reflections. The weighted *R*-factor *wR* and goodness of fit *S* are based on *F*^2^, conventional *R*-factors *R* are based on *F*, with *F* set to zero for negative *F*^2^. The threshold expression of *F*^2^ > σ(*F*^2^) is used only for calculating *R*-factors(gt) *etc*. and is not relevant to the choice of reflections for refinement. *R*-factors based on *F*^2^ are statistically about twice as large as those based on *F*, and *R*- factors based on ALL data will be even larger.

### Fractional atomic coordinates and isotropic or equivalent isotropic displacement parameters (Å^2^)

**Table:**

	*x*	*y*	*z*	*U*_iso_*/*U*_eq_	
C1	0.2534 (2)	0.22884 (14)	0.48554 (12)	0.0322 (4)	
C2	0.3341 (3)	0.15050 (17)	0.45355 (15)	0.0412 (5)	
H2	0.3794	0.1052	0.4887	0.049*	
C3	0.3457 (3)	0.14132 (19)	0.36796 (15)	0.0515 (6)	
H3	0.4008	0.0895	0.3457	0.062*	
C4	0.2783 (4)	0.2063 (2)	0.31554 (15)	0.0545 (6)	
H4	0.2864	0.1984	0.2582	0.065*	
C5	0.1984 (3)	0.28367 (18)	0.34767 (15)	0.0502 (6)	
H5	0.1530	0.3283	0.3119	0.060*	
C6	0.1848 (3)	0.29565 (16)	0.43303 (13)	0.0388 (5)	
H6	0.1304	0.3479	0.4547	0.047*	
C7	0.3242 (3)	0.32075 (14)	0.61007 (13)	0.0347 (4)	
C8	0.3275 (3)	0.32060 (16)	0.69897 (13)	0.0358 (4)	
C9	0.2629 (3)	0.24251 (15)	0.74015 (12)	0.0374 (4)	
C10	0.1679 (2)	0.17242 (14)	0.62462 (12)	0.0342 (4)	
C11	0.3990 (3)	0.38959 (15)	0.75391 (13)	0.0362 (4)	
C12	0.3869 (3)	0.36077 (15)	0.83528 (14)	0.0407 (5)	
C13	−0.0094 (3)	0.03243 (17)	0.62828 (15)	0.0483 (6)	
H13A	−0.1188	0.0260	0.6055	0.058*	
H13B	−0.0201	0.0488	0.6867	0.058*	
C14	0.0757 (4)	−0.0588 (2)	0.6213 (2)	0.0725 (9)	
H14A	0.0157	−0.1056	0.6532	0.087*	
H14B	0.1834	−0.0526	0.6462	0.087*	
C15	0.0958 (7)	−0.0951 (3)	0.5329 (3)	0.1005 (15)	
H15A	−0.0083	−0.1161	0.5121	0.151*	
H15B	0.1716	−0.1471	0.5325	0.151*	
H15C	0.1368	−0.0452	0.4981	0.151*	
C16	0.4758 (4)	0.47961 (18)	0.72536 (16)	0.0507 (6)	
H16A	0.3920	0.5227	0.7075	0.076*	
H16B	0.5486	0.4667	0.6798	0.076*	
H16C	0.5363	0.5074	0.7704	0.076*	
C17	0.4320 (3)	0.41350 (18)	0.91026 (15)	0.0443 (5)	
C18	0.4227 (5)	0.4121 (2)	1.05795 (16)	0.0689 (9)	
H18A	0.3688	0.4733	1.0586	0.083*	
H18B	0.5391	0.4218	1.0662	0.083*	
C19	0.3562 (5)	0.3512 (3)	1.12406 (18)	0.0761 (9)	
H19A	0.2411	0.3419	1.1151	0.114*	
H19B	0.3731	0.3809	1.1771	0.114*	
H19C	0.4110	0.2911	1.1231	0.114*	
N1	0.2447 (2)	0.24077 (12)	0.57496 (10)	0.0340 (4)	
N2	0.1836 (2)	0.16896 (13)	0.70577 (11)	0.0393 (4)	
N3	0.0749 (2)	0.10894 (14)	0.58518 (13)	0.0422 (4)	
H3A	0.055 (3)	0.121 (2)	0.5323 (18)	0.051*	
O1	0.3846 (2)	0.38072 (11)	0.56420 (9)	0.0453 (4)	
O2	0.4921 (3)	0.49108 (14)	0.91163 (11)	0.0578 (5)	
O3	0.3935 (3)	0.36454 (14)	0.97886 (11)	0.0609 (5)	
S1	0.29211 (8)	0.25028 (4)	0.84614 (3)	0.04848 (17)	

### Atomic displacement parameters (Å^2^)

**Table:**

	*U*^11^	*U*^22^	*U*^33^	*U*^12^	*U*^13^	*U*^23^
C1	0.0379 (9)	0.0327 (10)	0.0261 (9)	−0.0042 (7)	0.0009 (7)	0.0019 (7)
C2	0.0486 (12)	0.0380 (12)	0.0369 (11)	0.0050 (9)	−0.0041 (9)	0.0002 (9)
C3	0.0667 (15)	0.0487 (14)	0.0391 (12)	0.0029 (11)	0.0069 (11)	−0.0120 (10)
C4	0.0743 (17)	0.0613 (16)	0.0278 (10)	−0.0083 (14)	0.0003 (11)	−0.0038 (10)
C5	0.0619 (13)	0.0515 (13)	0.0372 (11)	−0.0084 (11)	−0.0076 (11)	0.0142 (11)
C6	0.0430 (11)	0.0356 (11)	0.0378 (11)	0.0019 (9)	−0.0006 (9)	0.0052 (8)
C7	0.0427 (11)	0.0287 (9)	0.0327 (10)	0.0026 (8)	0.0031 (8)	−0.0027 (8)
C8	0.0447 (11)	0.0326 (10)	0.0301 (10)	−0.0012 (9)	0.0029 (8)	−0.0024 (8)
C9	0.0489 (11)	0.0390 (11)	0.0242 (8)	0.0002 (10)	−0.0013 (8)	0.0001 (8)
C10	0.0439 (11)	0.0290 (10)	0.0297 (10)	−0.0004 (8)	0.0015 (8)	0.0028 (8)
C11	0.0412 (10)	0.0328 (10)	0.0344 (11)	0.0030 (8)	0.0017 (8)	−0.0056 (8)
C12	0.0498 (11)	0.0389 (11)	0.0334 (11)	−0.0011 (9)	0.0002 (9)	−0.0048 (9)
C13	0.0618 (14)	0.0451 (13)	0.0381 (12)	−0.0169 (11)	0.0044 (11)	0.0009 (10)
C14	0.079 (2)	0.0551 (17)	0.083 (2)	−0.0100 (15)	−0.0015 (17)	0.0234 (16)
C15	0.139 (4)	0.053 (2)	0.109 (3)	−0.005 (2)	0.043 (3)	−0.018 (2)
C16	0.0734 (17)	0.0381 (12)	0.0406 (13)	−0.0103 (11)	0.0032 (11)	−0.0060 (10)
C17	0.0517 (12)	0.0460 (13)	0.0354 (11)	0.0043 (10)	−0.0032 (10)	−0.0104 (10)
C18	0.104 (2)	0.0697 (19)	0.0329 (13)	−0.0100 (18)	−0.0062 (14)	−0.0123 (12)
C19	0.101 (2)	0.086 (2)	0.0413 (15)	0.000 (2)	−0.0002 (16)	−0.0025 (15)
N1	0.0460 (8)	0.0304 (8)	0.0258 (8)	−0.0008 (7)	0.0016 (6)	0.0007 (6)
N2	0.0547 (11)	0.0356 (9)	0.0276 (8)	−0.0092 (8)	−0.0007 (8)	0.0024 (7)
N3	0.0570 (11)	0.0387 (10)	0.0309 (9)	−0.0101 (8)	−0.0060 (8)	0.0028 (8)
O1	0.0673 (10)	0.0378 (9)	0.0308 (8)	−0.0124 (8)	0.0093 (7)	0.0004 (6)
O2	0.0789 (12)	0.0515 (11)	0.0429 (10)	−0.0127 (9)	−0.0058 (9)	−0.0113 (8)
O3	0.0943 (14)	0.0570 (11)	0.0313 (9)	−0.0150 (10)	−0.0040 (9)	−0.0089 (8)
S1	0.0721 (4)	0.0474 (3)	0.0259 (2)	−0.0128 (3)	−0.0033 (2)	0.0012 (2)

### Geometric parameters (Å, °)

**Table:**

C1—C6	1.384 (3)	C12—S1	1.751 (2)
C1—C2	1.387 (3)	C13—N3	1.456 (3)
C1—N1	1.448 (2)	C13—C14	1.468 (4)
C2—C3	1.385 (3)	C13—H13A	0.9700
C2—H2	0.9300	C13—H13B	0.9700
C3—C4	1.362 (4)	C14—C15	1.519 (5)
C3—H3	0.9300	C14—H14A	0.9700
C4—C5	1.373 (4)	C14—H14B	0.9700
C4—H4	0.9300	C15—H15A	0.9600
C5—C6	1.386 (3)	C15—H15B	0.9600
C5—H5	0.9300	C15—H15C	0.9600
C6—H6	0.9300	C16—H16A	0.9600
C7—O1	1.226 (3)	C16—H16B	0.9600
C7—N1	1.420 (3)	C16—H16C	0.9600
C7—C8	1.429 (3)	C17—O2	1.201 (3)
C8—C9	1.390 (3)	C17—O3	1.339 (3)
C8—C11	1.439 (3)	C18—O3	1.457 (3)
C9—N2	1.343 (3)	C18—C19	1.471 (5)
C9—S1	1.723 (2)	C18—H18A	0.9700
C10—N2	1.311 (3)	C18—H18B	0.9700
C10—N3	1.335 (3)	C19—H19A	0.9600
C10—N1	1.401 (3)	C19—H19B	0.9600
C11—C12	1.373 (3)	C19—H19C	0.9600
C11—C16	1.490 (3)	N3—H3A	0.88 (3)
C12—C17	1.463 (3)		
			
C6—C1—C2	120.7 (2)	C13—C14—C15	114.8 (3)
C6—C1—N1	120.37 (18)	C13—C14—H14A	108.6
C2—C1—N1	118.93 (18)	C15—C14—H14A	108.6
C3—C2—C1	118.4 (2)	C13—C14—H14B	108.6
C3—C2—H2	120.8	C15—C14—H14B	108.6
C1—C2—H2	120.8	H14A—C14—H14B	107.6
C4—C3—C2	121.5 (2)	C14—C15—H15A	109.5
C4—C3—H3	119.2	C14—C15—H15B	109.5
C2—C3—H3	119.2	H15A—C15—H15B	109.5
C3—C4—C5	119.7 (2)	C14—C15—H15C	109.5
C3—C4—H4	120.1	H15A—C15—H15C	109.5
C5—C4—H4	120.1	H15B—C15—H15C	109.5
C4—C5—C6	120.5 (2)	C11—C16—H16A	109.5
C4—C5—H5	119.8	C11—C16—H16B	109.5
C6—C5—H5	119.8	H16A—C16—H16B	109.5
C1—C6—C5	119.2 (2)	C11—C16—H16C	109.5
C1—C6—H6	120.4	H16A—C16—H16C	109.5
C5—C6—H6	120.4	H16B—C16—H16C	109.5
O1—C7—N1	119.66 (19)	O2—C17—O3	123.5 (2)
O1—C7—C8	126.5 (2)	O2—C17—C12	125.6 (2)
N1—C7—C8	113.87 (18)	O3—C17—C12	110.8 (2)
C9—C8—C7	118.0 (2)	O3—C18—C19	107.5 (3)
C9—C8—C11	113.54 (18)	O3—C18—H18A	110.2
C7—C8—C11	128.3 (2)	C19—C18—H18A	110.2
N2—C9—C8	126.94 (19)	O3—C18—H18B	110.2
N2—C9—S1	121.50 (16)	C19—C18—H18B	110.2
C8—C9—S1	111.54 (16)	H18A—C18—H18B	108.5
N2—C10—N3	120.16 (19)	C18—C19—H19A	109.5
N2—C10—N1	123.25 (18)	C18—C19—H19B	109.5
N3—C10—N1	116.58 (18)	H19A—C19—H19B	109.5
C12—C11—C8	110.74 (19)	C18—C19—H19C	109.5
C12—C11—C16	125.2 (2)	H19A—C19—H19C	109.5
C8—C11—C16	124.04 (19)	H19B—C19—H19C	109.5
C11—C12—C17	127.9 (2)	C10—N1—C7	121.80 (16)
C11—C12—S1	113.03 (16)	C10—N1—C1	120.45 (16)
C17—C12—S1	118.88 (17)	C7—N1—C1	117.67 (16)
N3—C13—C14	113.0 (2)	C10—N2—C9	115.30 (18)
N3—C13—H13A	109.0	C10—N3—C13	122.80 (19)
C14—C13—H13A	109.0	C10—N3—H3A	115.4 (19)
N3—C13—H13B	109.0	C13—N3—H3A	121.2 (19)
C14—C13—H13B	109.0	C17—O3—C18	116.2 (2)
H13A—C13—H13B	107.8	C9—S1—C12	91.12 (11)
			
C6—C1—C2—C3	0.6 (3)	S1—C12—C17—O3	1.2 (3)
N1—C1—C2—C3	−178.1 (2)	N2—C10—N1—C7	−10.4 (3)
C1—C2—C3—C4	−0.9 (4)	N3—C10—N1—C7	169.45 (18)
C2—C3—C4—C5	0.8 (4)	N2—C10—N1—C1	166.03 (19)
C3—C4—C5—C6	−0.5 (4)	N3—C10—N1—C1	−14.1 (3)
C2—C1—C6—C5	−0.3 (3)	O1—C7—N1—C10	−177.29 (19)
N1—C1—C6—C5	178.4 (2)	C8—C7—N1—C10	4.1 (3)
C4—C5—C6—C1	0.2 (4)	O1—C7—N1—C1	6.2 (3)
O1—C7—C8—C9	−175.2 (2)	C8—C7—N1—C1	−172.42 (17)
N1—C7—C8—C9	3.3 (3)	C6—C1—N1—C10	120.7 (2)
O1—C7—C8—C11	0.8 (4)	C2—C1—N1—C10	−60.5 (3)
N1—C7—C8—C11	179.28 (19)	C6—C1—N1—C7	−62.7 (3)
C7—C8—C9—N2	−6.1 (3)	C2—C1—N1—C7	116.0 (2)
C11—C8—C9—N2	177.4 (2)	N3—C10—N2—C9	−172.1 (2)
C7—C8—C9—S1	175.14 (16)	N1—C10—N2—C9	7.7 (3)
C11—C8—C9—S1	−1.4 (2)	C8—C9—N2—C10	0.5 (3)
C9—C8—C11—C12	0.4 (3)	S1—C9—N2—C10	179.13 (17)
C7—C8—C11—C12	−175.7 (2)	N2—C10—N3—C13	−1.6 (3)
C9—C8—C11—C16	−179.8 (2)	N1—C10—N3—C13	178.5 (2)
C7—C8—C11—C16	4.1 (4)	C14—C13—N3—C10	−100.7 (3)
C8—C11—C12—C17	−174.6 (2)	O2—C17—O3—C18	2.3 (4)
C16—C11—C12—C17	5.6 (4)	C12—C17—O3—C18	−176.2 (3)
C8—C11—C12—S1	0.8 (2)	C19—C18—O3—C17	173.9 (3)
C16—C11—C12—S1	−179.1 (2)	N2—C9—S1—C12	−177.32 (19)
N3—C13—C14—C15	−60.7 (4)	C8—C9—S1—C12	1.53 (17)
C11—C12—C17—O2	−2.1 (4)	C11—C12—S1—C9	−1.33 (19)
S1—C12—C17—O2	−177.2 (2)	C17—C12—S1—C9	174.47 (19)
C11—C12—C17—O3	176.3 (2)		

### Hydrogen-bond geometry (Å, °)

**Table:**

*D*—H···*A*	*D*—H	H···*A*	*D*···*A*	*D*—H···*A*
C6—H6···O2^i^	0.93	2.58	3.359 (3)	142
C2—H2···O2^ii^	0.93	2.50	3.432 (3)	177
N3—H3A···O1^iii^	0.88 (3)	2.08 (3)	2.863 (3)	147 (3)
C16—H16C···O2	0.96	2.31	3.000 (3)	128

Symmetry codes: (i) −*x*+1/2, −*y*+1, *z*−1/2; (ii) −*x*+1, *y*−1/2, −*z*+3/2; (iii) *x*−1/2, −*y*+1/2, −*z*+1.
